# Proceeding of the International Conference on Emerging Infectious Diseases 2000. Atlanta, Georgia, USA.

**DOI:** 10.3201/eid0707.010701

**Published:** 2001


### About the International Conference on Emerging Infectious Diseases 2000

**D. Peter Drotman,* Harold W. Jaffe,* Charles A. Schable,* Lori Feinman†**
*Centers for Disease Control and Prevention, Atlanta, Georgia, USA; and †American Society for Microbiology, Washington, DC, USA

Why would more than 2,000 epidemiologic, clinical, laboratory, veterinary, and other public health professionals from more than 70 nations gather in a very hot July in Atlanta, Georgia to discuss problems that vex the world? The International Conference on Emerging Infectious Diseases (ICEID) 2000 was the occasion, and they attended the conference because they are committed to working and learning together to make the world a safer and healthier place. The mission of preventing and responding to epidemics and epizootics represents a worthy challenge and tends to draw an eclectic partnership, which is well and good, for that is exactly what is necessary to accomplish this goal. More than 50 public and private, international and federal, academic and professional, charitable and corporate, and other organizations joined the partnership.

Atlanta was once again the site for the conference, which built upon its predecessor in 1998 (1). Plans are well under way for the third ICEID in March 2002 in Atlanta. Built around 12 plenary and 18 panel sessions, ICEID 2000 included more than 100 oral presentations, 300 poster presentations, four meet-the-professor/expert sessions, special sessions on bioterrorism and newsmedia coverage of health stories, an opening session that featured speakers James Hughes, from the Centers for Disease Control and Prevention, Senator Bill Frist, David Heymann, from the World Health Organization, Enriqueta Bond, from the Burroughs Wellcome Fund, and George Lundberg, editor-in-chief of Medscape, along with a closing session on West Nile virus encephalitis. Clearly, the amount of information provided was more than most participants could absorb. To assist them, as well as their colleagues around the world who could not attend, ICEID 2000 has been made available on the Internet. Audio and visual access is available for most of the plenary sessions, many of the panel sessions, and the opening and closing general sessions. Many PowerPoint slide presentations, graciously donated by the presenters, are also posted. (http://www.cdc.gov/iceid/)

Coincidentally, the same week that ICEID 2000 took place, the British Medical Journal published an article in its ongoing series on medical careers on communicable disease control, calling it “arguably the most successful specialty of all” (2). ICEID attendees and readers of Emerging Infectious Diseases can certainly relate to the list of pros and cons Dr. Sarah Woodhouse listed in the article:

Address for correspondence: Peter Drotman, Centers for Disease Control and Prevention, 1600 Clifton Road, Mailstop C12, Atlanta, GA 30333, USA; fax: 404-639-3039; e-mail: dpd1@cdc.gov

### Advantages

- Dynamic nature of work. Communicable diseases are constantly adapting and evolving.
- Diversity. Disease control is both reactive and proactive work.
- Multidisciplinary. Health professionals have increasing opportunities to work in teams with a variety of other professionals.
- Flexible. The on-call commitment rarely interferes with normal activities.
- Opportunities for career development. Regional epidemiology work, research, and teaching are just a few examples of professions relating to communicable diseases.

### Disadvantages

- Communicable disease control function is still underresourced in many areas.
- The role of health-care workers in disease prevention is often poorly understood by medical and other colleagues.
- Communicable disease control offers limited opportunities for lucrative private work.

Many persons in the public health community can clearly see how these issues influence the local and global effots to prevent infectious disease emergence and reemergence. We may, in part, be victims of our own recent and past successes. If the price of liberty is eternal vigilance, similarly the price of a world free of plagues is eternal surveillance and appropriate response. Perhaps the most important lessons from ICEID 2000 are that there is no reason for any of us to relax our efforts and that the need for ICEID will continue for a long time to come.

### Acknowledgments

The organizers of the International Conference on Emerging Infectious Diseases 2000 thank Carol Snarey, Kelly Holton, and Margaret Songe for their assistance in editing the conference presentations.

### References

1. Morse S. About the International Conference on Emerging Infectious Diseases. Emerg Infect Dis 1998;4:353.
2. Woodhouse S. Career focus: communicable disease control. BMJ [serial online] 2000 [cited 2001 May 10];321:S2. (This article is listed in the table of contents of the print version of the BMJ but is found only in the online version: http://bmj.com/cgi/content/full/321/7254/S2-7254)

